# Transient Sperm Starvation Improves the Outcome of Assisted Reproductive Technologies

**DOI:** 10.3389/fcell.2019.00262

**Published:** 2019-11-05

**Authors:** Felipe A. Navarrete, Luis Aguila, David Martin-Hidalgo, Darya A. Tourzani, Guillermina M. Luque, Goli Ardestani, Francisco A. Garcia-Vazquez, Lonny R. Levin, Jochen Buck, Alberto Darszon, Mariano G. Buffone, Jesse Mager, Rafael A. Fissore, Ana M. Salicioni, María G. Gervasi, Pablo E. Visconti

**Affiliations:** ^1^Department of Veterinary and Animal Sciences, Integrated Sciences Building, University of Massachusetts Amherst, Amherst, MA, United States; ^2^Research Group of Intracellular Signaling and Technology of Reproduction, Institute of Biotechnology in Agriculture and Livestock (INBIO G + C), University of Extremadura, Cáceres, Spain; ^3^Instituto de Biología y Medicina Experimental, Consejo Nacional de Investigaciones Científicas y Técnicas, Buenos Aires, Argentina; ^4^Department of Physiology, Veterinary School, International Excellence Campus for Higher Education and Research, University of Murcia, Murcia, Spain; ^5^Institute for Biomedical Research of Murcia, IMIB-Arrixaca, Murcia, Spain; ^6^Department of Pharmacology, Weill Cornell Medical College, Cornell University, New York, NY, United States; ^7^Departamento de Genética del Desarrollo y Fisiología Molecular, Instituto de Biotecnología, Universidad Nacional Autónoma de México, Cuernavaca, Mexico

**Keywords:** sperm, blastocyst, embryo transfer, IVF, ICSI, capacitation

## Abstract

To become fertile, mammalian sperm must undergo a series of biochemical and physiological changes known as capacitation. These changes involve crosstalk between metabolic and signaling pathways and can be recapitulated *in vitro*. In this work, sperm were incubated in the absence of exogenous nutrients (starved) until they were no longer able to move. Once immotile, energy substrates were added back to the media and sperm motility was rescued. Following rescue, a significantly higher percentage of starved sperm attained hyperactivated motility and displayed increased ability to fertilize *in vitro* when compared with sperm persistently incubated in standard capacitation media. Remarkably, the effects of this treatment continue beyond fertilization as starved and rescued sperm promoted higher rates of embryo development, and once transferred to pseudo-pregnant females, blastocysts derived from treated sperm produced significantly more pups. In addition, the starvation and rescue protocol increased fertilization and embryo development rates in sperm from a severely sub-fertile mouse model, and when combined with temporal increase in Ca^2+^ ion levels, this methodology significantly improved fertilization and embryo development rates in sperm of sterile CatSper1 KO mice model. Intracytoplasmic sperm injection (ICSI) does not work in the agriculturally relevant bovine system. Here, we show that transient nutrient starvation of bovine sperm significantly enhanced ICSI success in this species. These data reveal that the conditions under which sperm are treated impact post-fertilization development and suggest that this “starvation and rescue method” can be used to improve assisted reproductive technologies (ARTs) in other mammalian species, including humans.

## Introduction

Sperm capacitation is defined as all the biochemical and physiological events occurring after epididymal maturation that are necessary for the sperm to acquire fertilizing capacity (Yanagimachi, 1994; Gervasi and Visconti, 2016). Following this definition, capacitation ends once the sperm fuses with a metaphase II (MII)-arrested egg to become a zygote. The zygote follows a series of complex cell biological processes leading to the first cleavage, continuing with pre-implantation development and differentiation, implantation, and finally, birth of an offspring (White et al., 2018). Among these processes, capacitation, fertilization, and early embryo development can be mimicked *in vitro* in defined media (Ventura-Junca et al., 2015). Fertilization can also be achieved by intracytoplasmic sperm injection (ICSI) effectively bypassing many physiological events preceding gamete fusion (Palermo et al., 2017). These techniques are well established in many mammalian species including humans. However, the efficiency of these methods is species-specific, and success depends on other factors including the age of male and female gametes, environmental factors, and genetic background (Meldrum et al., 2016). Although many problems in capacitation can be overcome using ICSI, obtaining good quality preimplantation embryos at the blastocyst stage is a major limiting factor of successful pregnancies (Sadeghi, 2017).

Sperm capacitation depends upon crosstalk between metabolic and signaling pathways (Goodson et al., 2012). In mouse sperm, changes in motility pattern associated with capacitation (i.e., hyperactivation) are based on ATP produced by glycolysis (Miki et al., 2004). However, it is also clear that the Krebs cycle and oxidative phosphorylation are active in these cells (Goodson et al., 2012) and mammalian sperm capacitation media contain fuels for both glycolysis (i.e., glucose) and oxidative phosphorylation (i.e., pyruvate). To explore the specific role of these fuels for capacitation, initially, we incubated mouse sperm in media devoid of all nutrients (starvation step). Once sperm became motionless, defined energy molecules (e.g., glucose and pyruvate) were added back (rescue step). We hypothesized that after energy depletion, adding back nutrients to the sperm incubation media would induce recovery of sperm functionality. Indeed, the recovery was observed; however, the starvation and rescue protocol significantly increased the percentage of sperm achieving hyperactivated motility and also enhanced *in vitro* fertilization (IVF) rates when compared with sperm persistently incubated in standard capacitation media containing glucose and pyruvate. Also unexpectedly, the embryos fertilized with previously starved and recovered sperm were more efficient in reaching the blastocyst stage; and these blastocysts produced approximately three times more pups when transferred to pseudo-pregnant females.

The need for capacitation is often overcome by the use of ICSI. Although this procedure is highly successful in mice, ICSI with bovine sperm presents severe challenges and only succeeds when the egg is artificially activated (Catt and Rhodes, 1995; Malcuit et al., 2006). We now show that the starvation and rescue protocol improves ICSI success rates in the bovine system. Together, these results indicate that sperm incubation conditions can improve the outcome of embryo development and that capacitation-associated changes occurring in sperm before fertilization are still relevant for post-fertilization events. The molecular basis of these changes is still not known and warrants future investigation. Some of the hypotheses regarding these mechanisms will be briefly mentioned in the section “Discussion.”

## Results

**FIGURE 1:**
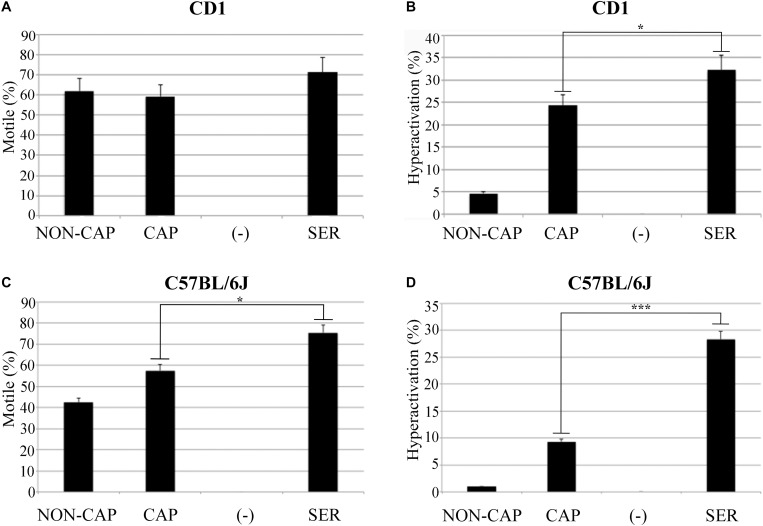
Sperm energy restriction and recovery (SER) treatment improves sperm motility. Experimental conditions non-capacitated (NON-CAP), capacitated (CAP), sperm energy restriction without recovery (-) or sperm energy restriction and recovery (SER) were established by the incubation in different media as indicated in the section “Materials and Methods.” After treatment under the different conditions, sperm motility was analyzed by computer-assisted sperm analysis (CASA). Two different strains of mice were analyzed, as follows: **(A)** Percentage of total motility for sperm of the outbred mice strain CD1. **(B)** Percentage of hyperactivated motility (out of total motile) for sperm of the outbred mice strain CD1. **(C)** Percentage of total motility for sperm of the inbred mouse strain C57BL/6J. **(D)** Percentage of hyperactivated motility (out of total motile) for sperm of the inbred mouse strain C57BL/6J. In each panel, significant differences are indicated as: ^∗^*p* < 0.05 and ^∗∗∗^*p* < 0.001, *n* = 5.

Sperm from CD1 mice incubated in TYH media lacking glucose and pyruvate stopped moving after ∼40 min (Supplementary Video S1). We reasoned that these sperm had ceased swimming because they had consumed all available stored nutrients, and we refer to them as “starved.” Once immotile, glucose and pyruvate were added back to reach standard concentrations in TYH media (5.56 mM glucose and 0.5 mM pyruvate, Supplementary Video S1). For the purpose of comparison, parallel control incubations were done resuspending sperm in standard TYH capacitating conditions persistently incubated in the presence of nutrients. Counter-intuitively, a higher percentage of starved and capacitated sperm were motile (Figure 1A) and became hyperactivated (Figure 1B) after addition of nutrients compared to control sperm persistently incubated in the presence of nutrients in standard TYH capacitating conditions. Hereafter, we refer to this procedure, in which sperm energy is recovered after starvation, using the acronym SER.

**FIGURE 2:**
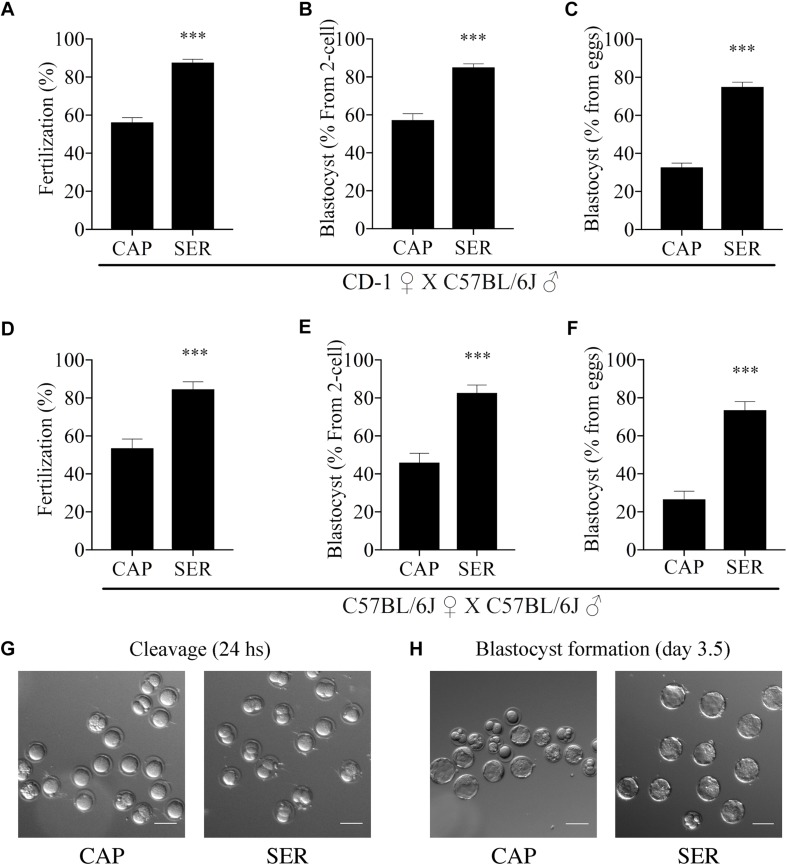
Sperm energy restriction and recovery (SER) treatment improves fertilization and embryo development rates in C57BL/6J male mice. Sperm obtained from C57BL/6J mice were incubated in C-TYH, containing glucose and pyruvate (CAP) or in F-TYH media, not containing nutrients; sperm in this medium will then be rescued by energy sources replenishment as explained in the section “Materials and Methods,” and the treatment abbreviated (SER). After treatment (CAP or SER) sperm from each condition were added to the insemination drops containing cumulus-enclosed female oocytes (COCs). **(A)** Percentage of fertilization calculated as percentage of oocytes that reached a two-cell embryo stage after heterologous fertilization between CD-1 female oocytes and C57BL/6J sperm (*n* = 38, total number of oocytes CAP = 2030, total number of oocytes SER = 2024). **(B)** Percentage of blastocyst development calculated as percentage of two-cell embryos that reached 3.5-day blastocyst stage corresponding to embryo cultures after heterologous CD-1 female oocytes and C57BL/6J sperm IVF (*n* = 38). **(C)** Percentage of total blastocyst development calculated as the percentage of 3.5-day blastocyst stage embryos out of the total number of inseminated eggs corresponding to embryo cultures after heterologous CD-1 female oocytes and C57BL/6J sperm IVF (*n* = 38). **(D)** Percentage of fertilization calculated as percentage of oocytes that reached two-cell embryo stage after homologous fertilization between C57BL/6J female oocytes and C57BL/6J sperm (*n* = 15, total number of oocytes CAP = 608, total number of oocytes SER = 685). **(E)** Percentage of blastocyst development calculated as percentage of the two-cell embryos that reached 3.5-day blastocyst stage from embryo cultures after homologous C57BL/6J female oocytes and C57BL/6J sperm IVF (*n* = 15). **(F)** Percentage of total blastocyst development calculated as the percentage of 3.5-day blastocyst stage embryos out of the total number of inseminated eggs from embryo cultures after homologous C57BL/6J female oocytes and C57BL/6J sperm IVF (*n* = 15). In each panel, significant differences are indicated as ^∗∗∗^*p* < 0.001. **(G)** Representative images of two-cell embryos obtained after heterologous, CD-1 female oocytes and C57BL/6J sperm, SER, or control IVF. Scale bar = 100 μm. **(H)** Representative images of heterologous IVF-derived blastocyst development at 3.5 days. Scale bar = 100 μm.

Considering that SER produced a higher percentage of hyperactivated sperm, we hypothesized that SER-treated sperm would also improve fertilization rates. IVF using sperm from CD1 mice is highly efficient (over 90%). Therefore, to test the effectiveness of SER in IVF and other assisted reproductive technologies (ARTs), we switched to using sperm from inbred C57BL/6J which have lower IVF and embryo development rates (Liu et al., 2009). C57BL/6J sperm were capacitated in standard TYH media with BSA and HCO_3_^–^ (CAP) containing nutrients or incubated in nutrient-free CAP TYH media until becoming immotile and then rescued by addition of glucose and pyruvate (SER). Similar to sperm from CD1 mice, SER increased motility (Figure 1C) and hyperactivation rates (Figure 1D) of C57BL/6J sperm. In addition, SER-treated C57BL/6J sperm yielded more eggs reaching the two-cell stage when incubated with heterologous CD1 MII-arrested eggs (Figure 2A; *n* = 38) as well as when incubated with homologous MII-arrested eggs from C57BL/6J females (Figure 2D; *n* = 15). Representative images of two-cell stage embryos are displayed in Figure 2G.

**FIGURE 3:**
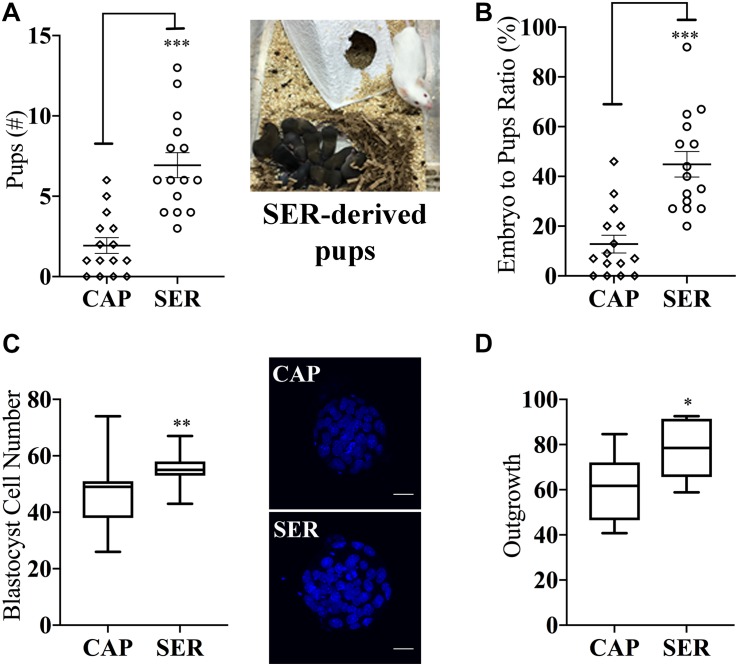
Sperm energy restriction and recovery (SER) treatment impacts on embryonic parameters and implantation potential after non-surgical embryo transfer. A fraction of the blastocysts derived from CAP or SER treatments as described in Figure 2 were used to evaluate the impact of these treatments in blastocyst potential. **(A)** Blastocysts derived from CAP- or SER-treated sperm were transferred to pseudo-pregnant recipient female mice. The total number of pups obtained from either CAP or SER treatment (*n* = 15) is indicated. Image shown is representative of a litter obtained after transfer of SER-derived embryos. **(B)** Percentage of pups born per number of embryos transferred is indicated. **(C)** Blastocysts derived from CAP- or SER-treated sperm were stained with Hoechst as indicated in the section “Materials and Methods.” The number of stained nuclei that represents the number of cells in each blastocyst was counted (*n* = 3, 35 blastocysts/treatment counted). Representative images of stained embryos are shown. Scale bar = 25 μm. **(D)** Blastocyst derived from CAP or SER-treated sperm were assayed for outgrowth *in vitro* as explained in the section “Materials and Methods” (*n* = 10, 205 and 210 blastocysts counted for CAP and SER-derived blastocysts, respectively). In each panel, significant differences are indicated as: ^∗^*p* < 0.05; ^∗∗^*p* < 0.01, and ^∗∗∗^*p* < 0.001. Experiments represented in this figure were done using blastocysts obtained by heterologous fertilization.

More surprising than the improvement observed in fertilization rates, in both homologous and heterologous conditions, a significantly higher percentage of two-cell embryos reached blastocyst stage (Figures 2B,E blastocyst % from two-cell). Representative images of blastocyst development are shown in Figure 2H. To compare the efficiency of the treatments, these results were also presented as the percentage of the initial number of eggs reaching blastocyst stage (Figures 2C,F, blastocyst % from initial number of eggs). For more information, these data are also presented in Supplementary Figure S1 and tabulated in Supplementary Table S1 according to male mice age. To evaluate blastocyst developmental potential, in a fraction of the experiments described above (*n* = 19, *n* = 15 for heterologous CD1 female x C57BL/6J male IVF and *n* = 4 for homologous C57BL/6J female x C57BL/6J male), an equal number of blastocysts derived from CAP or SER-treated sperm were non-surgically transferred to pseudo-pregnant females. Unexpectedly, the number of pups born from SER-treated sperm derived blastocysts was over three times higher than the number of pups born from control blastocysts (Figure 3A for heterologous IVF and Supplementary Table S1 for heterologous and homologous IVF). The improvement in embryo development is reflected in the increased ratio of transferred blastocysts producing offspring (Figure 3B and Supplementary Table S1). In addition, blastocyst quality was also assessed by counting the total cell number present at the blastocyst stage (Figure 3C) as well as by blastocyst outgrowth (Figure 3D). These analyses revealed a higher number of cells and percentage of outgrowth of blastocysts derived from SER-treated sperm. Thus, these data indicate that transient nutrient withdrawal provides a qualitative improvement in the ability of sperm to produce embryos, and this effect is not simply due to increased ability to fertilize oocytes.

**FIGURE 4:**
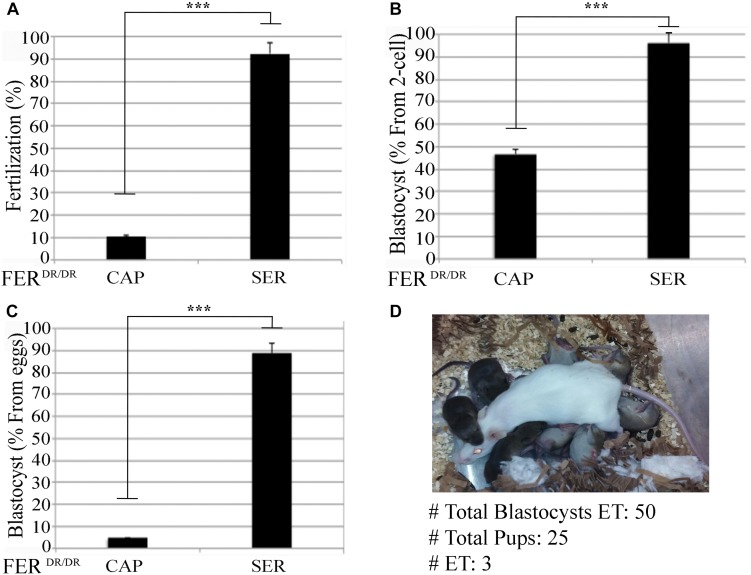
Sperm energy restriction and recovery (SER) treatment rescues sperm fertilization capacity and embryo development of a sub-fertile mouse strain. Sperm obtained from Fer^DR/DR^ mice were treated under CAP or SER conditions and used for insemination as described in the section “Materials and Methods.” **(A)** Percentage of fertilization of Fer^DR/DR^ sperm calculated as percentage of oocytes that reached two-cell embryo stage. **(B)** Percentage of blastocyst development calculated as percentage of the two-cell embryos that reached 3.5-day blastocyst stage. **(C)** Percentage of total blastocyst formation calculated as the percentage of 3.5-day blastocyst stage embryos out of the total number of inseminated eggs. **(D)** Representative image of a recipient female with its embryo-transferred pups. A total of three embryo transfers (ET) were performed as indicated in Supplementary Table S1. In each panel, significant differences are indicated as ^∗∗∗^*p* < 0.001.

**FIGURE 5:**
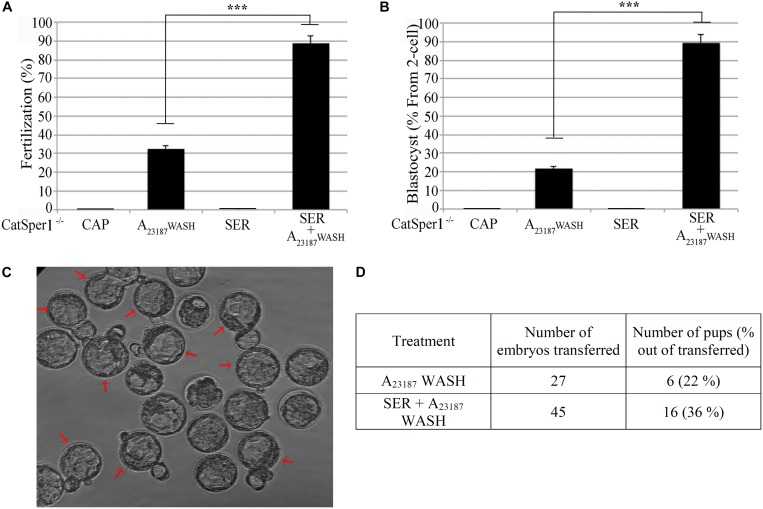
Combination of sperm energy restriction and recovery (SER) treatment and A23187 (transient treatment) rescues sperm fertilization capacity and embryo development of infertile CatSper1 KO mice. Sperm from CatSper1 KO mice were incubated in four conditions as detailed in the section “Materials and Methods.” Briefly, the experimental conditions applied were: (1) capacitating conditions in the presence of glucose and pyruvate (CAP); (2) A23187 transient treatment (A23187 WASH); (3) capacitating conditions in the absence of glucose and pyruvate followed by replenishment of energy substrates (SER); and (4) SER treatment followed by A23187 transient treatment (SER + A23187 WASH). Sperm were used for IVF of cumulus-enclosed CD1 female oocytes. After 18 h, two-cell embryos were transferred to KSOM media and further incubated for a total of 3.5 days until blastocyst stage. **(A)** Percentage of fertilization calculated as percentage of oocytes that reached two-cell embryo stage. **(B)** Percentage of blastocyst development calculated as percentage of the two-cell embryos that reached 3.5-day blastocyst stage. **(C)** Representative image of 3.5-day blastocysts formation from combined SER treatment followed by A23187 transient treatment. Arrows indicate blastocysts hatching. **(D)** Results showing total 3.5-day blastocysts obtained from either SER-treated alone, or SER + A23187-treated sperm, that were NSET-transferred to pseudo-pregnant female recipients (*n* = 3), and the number of pups born after 21 days of gestation. In each panel, significant differences are indicated as ^∗∗∗^*p* < 0.001.

The aforementioned experiments indicate an overall better success in fertilization, embryo development, and pregnancy rates when SER-treated sperm are used. We next sought to determine if this methodology enhances IVF and embryo development rates in sub-fertile and sterile mouse models. Recently, we showed that sperm derived from FER^DR/DR^ mice, in which the FER tyrosine kinase is replaced by a non-active mutant, are severely sub-fertile *in vitro* (Alvau et al., 2016). When compared to sperm incubated in standard capacitation media, SER-treated FER^DR/DR^ sperm significantly increased fertilization rates (Figure 4A) and embryo development (Figures 4B,C). When these embryos were transferred to pseudo-pregnant females, live pups were born (Figure 4D). We next tested the SER treatment on sperm from CatSper1 KO mice, which are sterile (Ren et al., 2001). We recently found that a short incubation of CatSper1 KO sperm with Ca^2+^ ionophore A_23187_ induced fertilizing capacity in these normally infertile sperm (Navarrete et al., 2016). SER treatment alone did not rescue the fertilizing capacity of CatSper1 KO sperm. However, the two treatments proved to be synergistic; sequential treatment of CatSper1 KO sperm with SER and A_23187_ significantly increased fertilization (Figure 5A) and embryo development (Figure 5B) rates when compared with A_23187_ alone. When blastocysts (Figure 5C) from these experiments were transferred to pseudo-pregnant females, live pups were born (Table in Figure 5D).

While ICSI in the mouse is quite successful, the efficiency of this technique in bovines is very poor when compared to other species (Salamone et al., 2017). Therefore, we used frozen bovine sperm and *in vitro* matured bovine oocytes to test if SER can also improve ICSI success rates. Contrary to mouse sperm, bovine sperm motility was reduced but did not completely stop during incubation in the absence of exogenous energy sources. Bovine capacitation media normally contain pyruvate and lactate but do not contain glucose; therefore, after 3 h in the absence (starvation step) of nutrients, pyruvate and lactate were added back, and normal motility was restored. Individual bovine sperm were injected into oocytes (ICSI) and the number of zygotes undergoing cleavage divisions was evaluated. The percentage of two-cell embryos was increased over threefold in SER-treated bovine sperm relative to controls persistently incubated in the presence of nutrients. Importantly, almost 17% of these two-cell embryos developed into blastocysts compared to zero blastocysts obtained from sperm incubated in standard capacitation conditions (Table 1).

**TABLE 1:** Effect of SER treatment on the *in vitro* development of bovine embryos generated by ICSI, piezoelectric-aided.

**Treatment**	***n* (total)**	**Day 3 cleaved embryos (%)**	**Day 8 Blastocyst/cleaved embryos (%)**
ICSI control	5 (80)	13 (16)^b^	0 (0)^b^
ICSI + SER	5 (84)	42 (50)^a^	7 (17)^a^

*Data followed by different superscripts (a,b) are significantly different (*p* < 0.05).*

## Discussion

Infertility and sub-fertility are critical health problems with social and economic consequences. About 9% of couples worldwide present signs of infertility. Since the first successful “Test-Tube” baby in 1978, over 5 million babies were born using ARTs. ARTs include IVF, artificial insemination (AI), ICSI, *in vitro* maturation of oocytes, superovulation, cryopreservation, and embryo transfer techniques. In addition to their use in human clinical application, ARTs are also used for agricultural domestic species and for production and maintenance of valuable laboratory animals which often present reproductive problems (Behringer et al., 2014). Despite their frequent use, ART methods are usually expensive, time consuming, and can be plagued by limited successful pregnancy rates (de Mouzon et al., 2010; Mantikou et al., 2013). At the basic research level, our group has recently shown that we can induce *in vitro* fertilizing capacity in sperm from sterile knock-out genetic models using a short incubation with the Ca^2+^ ionophore A_23187_ (Navarrete et al., 2016). Interestingly, short treatment with A_23187_ also increased fertilization and embryo development rates of control inbred C57BL/6J mice sperm (Navarrete et al., 2016) suggesting that embryo development can be affected by differential sperm incubation conditions during capacitation.

Capacitation can be achieved *in vitro* in defined media containing ions, a cholesterol binding source, and energy substrates (reviewed in Gervasi and Visconti (2016)). In the mouse, capacitation and IVF can be achieved with TYH medium containing only glucose and pyruvate as energy compounds (Toyoda and Yokoyama, 2016). Here, we observed that mouse sperm incubated in the absence of these exogenous nutrients become immotile in less than 40 min and motility can be rescued with the addition of glucose and pyruvate. Once rescued, starved sperm achieved higher percentages of hyperactivation and IVF rates. We do not yet know the molecular basis for this improvement in sperm functional parameters. Two alternative possibilities to explain these results are that due to the need for energy sources molecules limiting capacitation competence are consumed during the starvation stage; or that during rescue, molecules supporting capacitation are upregulated. Another possibility is that the starvation followed by rescue protocol induces changes in metabolites, supporting an increase of certain metabolites that could be beneficial for the development of sperm hyperactivated motility and fertilization. Finally, we cannot discard changes in the production of radical oxygen species (ROS) which have been associated with deleterious effects on sperm motility as well as with beneficial effects on capacitation (de Lamirande and Gagnon, 1995; Aitken, 2017). Further investigations are being performed to test these hypotheses.

Unexpectedly, eggs fertilized with SER-treated sperm showed significantly greater percentage of two-cell embryos reaching the blastocyst stage. Moreover, at 3.5 days post-fertilization, blastocysts derived from SER-treated sperm had a higher percentage of outgrowth and higher number of total cells, parameters that are indicative of good implantation potential (Binder et al., 2015). Furthermore, when transferred to pseudo-pregnant females, blastocysts derived from SER-treated sperm gave rise to a higher number of pups than those obtained with sperm incubated in standard capacitation conditions. These experiments indicate that blastocysts derived from SER-treated sperm have advantages over those obtained from sperm incubated in standard capacitation media. In concordance with our findings, it has been proposed that sperm incubation conditions for IVF can affect embryonic development (Jaakma et al., 1997; Matas et al., 2003; Zheng et al., 2018). However, the molecular basis of sperm-derived post-fertilization effects remains unknown. We hypothesize that the post-fertilization effects observed after sperm SER treatment are related to epigenetic changes in the male gamete. Environmental factors can influence epigenetic processes, change gene expression, and have a great impact on development. Epigenetic marks such as, DNA methylation, and histone tail modifications, show asymmetrical distribution between male and female gamete and play critical roles in embryo development (Marcho et al., 2015; Canovas et al., 2017). Recent evidence suggests that small non-coding RNAs in sperm have a role in non-genomic inheritance of paternal traits (Chen et al., 2016; Sharma et al., 2016; Conine et al., 2018). One intriguing possibility is that SER treatment can affect RNA levels including the pool of non-coding RNAs, which could impact post-fertilization development. On the other hand, it has been shown that the state of the sperm chromatin contains critical information for the regulation of gene expression after fertilization (Jung et al., 2017, 2019). One possibility is that SER treatment is inducing changes in the sperm chromatin prior fertilization, and that those changes have a direct impact in the development of the embryo. We are currently studying these possibilities.

Intracytoplasmic sperm injection is a technique by which sperm are directly introduced into the egg, bypassing the need for capacitation. In mice, ICSI is very successful, effectively rescuing several sperm sterile phenotypes (Kuretake et al., 1996; Yanagimachi et al., 2004; Ihara et al., 2005). In contrast, embryo development after ICSI is compromised in bovines, and successful development requires additional treatments with reagents that induce egg activation (Catt and Rhodes, 1995; Malcuit et al., 2006; Salamone et al., 2017). ICSI has also been achieved by pre-treating bovine sperm or eggs with diverse reagents (Rho et al., 1998; Aguila et al., 2017; Canel et al., 2017). At present, it is not clear why bovine sperm do not induce egg activation, but our data suggest that SER treatment of bovine sperm stimulates their ability to activate oocytes. One of the best characterized sperm contributions to early embryo development is the phospholipase C zeta (PLCζ)-mediated induction of Ca^2+^ oscillations (Saunders et al., 2002). We hypothesized that in bovine sperm, the PLCζ protein and its activity are closely guarded such that its release requires active nuclear remodeling. Consistently, our group has recently shown that injected bovine sperm are resistant to nuclear remodeling and decondensation by *in vitro* matured bovine oocytes and that treating these oocytes with ionomycin plus cycloheximide (CHX) to chemically induce acute activation and sperm head decondensation can partially overcome the poor success of this technique in this species (Aguila et al., 2017). In addition, it has been recently shown that the sperm-borne protein glutathione-S-transferase omega 2 facilitates nuclear decondensation after fertilization (Hamilton et al., 2019). These results suggest that during penetration through the cumulus and zona pellucida layers, bovine sperm undergo changes that induce or support nuclear remodeling, which facilitate the subsequent exposure and/or activation of PLCζ. Our data indicate that SER treatment of bovine sperm stimulates their ability to activate oocytes following fertilization by ICSI. We hypothesize that in bovine sperm, SER conditions induce remodeling of the sperm head facilitating nuclear decondensation and PLCζ release, just as it occurs during the process of natural sperm entry and fertilization. It will be interesting to determine whether the mechanism increasing sperm functional parameters and improving embryo development success rates in mice are related to the molecular changes which increase egg activation rates in bovines.

In all ARTs, the limiting step for a successful pregnancy is the attainment of embryos competent for implantation that can develop into viable fetuses (Geary and Moon, 2006; Patrizio et al., 2007). Poor quality of embryos results in higher rates of implantation failure (Takeuchi et al., 2017). Not surprisingly, most clinical research in humans and species of economic relevance has focused on improving embryo culture techniques (Summers, 2013) and selecting the best embryos to be transferred to the female (Castello et al., 2016; Vaiarelli et al., 2016). In particular, embryos can be selected by monitoring embryo development in real time using imaging technology or by preimplantation genetic diagnostic (PGD; Castello et al., 2016; Vaiarelli et al., 2016). In comparison, less research has been conducted to investigate sperm contributions to embryo quality. For ICSI, recent efforts focused on selecting high quality sperm using high microscopy magnification (Goswami et al., 2018), a method, known as morphologically selected sperm injection (IMSI). IMSI has been used by different clinics with a variety of outcomes (De Vos et al., 2013; Luna et al., 2015). More recently, thermotaxis was used to select sperm for ICSI using temperature gradients (Perez-Cerezales et al., 2018). After ICSI, thermotaxis-selected sperm resulted in higher percentage of blastocyst development and better implantation rates than non-selected sperm (Perez-Cerezales et al., 2018). These investigations suggest that the condition of the sperm prior to fertilization contributes to embryo development and that sperm selection prior to ICSI has the potential to be used in the clinic to improve pregnancy rates. Similarly, SER-treated sperm increased embryo development and pregnancy rates; however, instead of selecting the best sperm, SER appears to improve the overall sperm quality increasing their chances to produce high-quality embryos.

Translationally, SER treatment can be used for more efficient transgenic mouse production. SER treatment increased fertilization and embryo development rates of Fer^DR/DR^ mice which have a severely *in vitro* sub-fertile phenotype (Alvau et al., 2016). As another model, CatSper1 KO mice are infertile (Ren et al., 2001). In previous work, we showed that a short exposure of Ca^2+^ ionophore A_23187_ to sperm induced IVF rates of 30% in CatSper1 KO mice (Navarrete et al., 2016). SER treatment alone was not able to induce fertilizing capacity in sperm from sterile CatSper1 KO mice. One important difference between these two models is that while FER^DR/DR^ mouse model is fertile *in vivo*, CatSper1 KO is completely sterile. We hypothesize that SER treatment is able to bypass lack of tyrosine phosphorylation in FER^DR/DR^ because sperm from these mice are still able to fertilize. In this case, SER is only improving their ability to fertilize. On the other hand, CatSper1 KO mice are completely sterile *in vivo* and *in vitro*. When we combined both treatments, we found a synergistic increase of fertilization and embryo development rates in CatSper1 KO mice. These experiments suggest that an increase in sperm [Ca^2+^]_i_ is still required for SER to work.

Overall, the sperm energy restriction treatment is a novel method that improves both sperm fertilization rates and embryo development after IVF. The molecular mechanisms triggered by the sperm SER treatment prior IVF or ICSI are still not understood. Our data raise a series of very important still unanswered questions that call for new investigations in the field of reproductive biology and early embryo development. Importantly, our results indicate that sperm capacitation conditions continue to be relevant after fertilization has occurred, opening new avenues to study the relationship between sperm capacitation and early embryo development. The overall SER improvements in sperm function before and after fertilization have the potential to be applied to other mammalian species including humans and to impact ART practices worldwide.

## Materials and Methods

### Materials

Chemicals and other lab reagents were purchased as follows: 4-Bromo-Ca^2+^ Ionophore A_23187_ (B7272), bovine serum albumin (BSA, fatty acid-free, A0281) for media, pregnant mare serum gonadotropin (PMSG) (G4877), and human chorionic gonadotropin (hCG) (CG5) from Sigma (St. Louis, MO, United States). Non-surgical embryo transfer (NSET) device was acquired from Paratechs (Billerica, MA, United States). Light mineral oil (ES-005-C) and EmbryoMax^®^ KSOM Medium (1X) with 1/2 Amino Acids (MR-106-D) were obtained from Millipore (Billerica, MA, United States). Paraformaldehyde was purchased from Electron Microscopy Sciences (Hatfield, PA, United States). Hoechst 33342 was obtained from ThermoFisher Scientific (Agawam, MA, United States).

### Animals

All procedures involving experimental animals were performed in accordance with Protocol 2016-0026 approved by the University of Massachusetts Amherst Institutional Animal Care and Use Committee (IACUC). Male CD-1 (ICR) retired breeders, female CD1 (ICR) 8–12 weeks of age, and male CD-1 (ICR) vasectomized mice were obtained from Charles River Laboratories (Wilmington, MA, United States). Male C57BL/6J (different ages) and female C57BL/6J 8-weeks of age mice were obtained from The Jackson Laboratory (Farmington, CT, United States). Male Fer ^DR/DR^ mice (Craig et al., 2001) were obtained from Dr. Greer from Queen’s University, Kingston (ON, Canada). Infertile CatSper1 KO (Ren et al., 2001) mice were obtained from Dr. Clapham and Dr. Chung from Harvard University, Boston (MA, United States).

### Computer-Assisted Sperm Analysis (CASA)

Sperm incubation for analysis of motility was performed in a HEPES modified Toyoda–Yokoyama–Hosi (HEPES-TYH) medium (Toyoda et al., 1971) containing 119.37 mM NaCl, 4.7 mM KCl, 1.71 mM CaCl_2_.2H_2_O, 1.2 mM KH_2_PO_4_, 1.2 mM MgSO_4_.7H_2_O, 0.51 mM Na-pyruvate, 5.56 mM glucose, and 20 mM HEPES (pH 7.4). This medium does not contain HCO_3_^–^ or BSA and does not support capacitation; it was named (NON-CAP-HEPES-TYH) and abbreviated as NON-CAP in Figure 1. The same medium supplemented with 15 mM HCO_3_^–^ and 5 mg/mL BSA support capacitation, it was named CAP-HEPES-TYH and abbreviated as CAP in Figure 1. Starvation was done in CAP medium in which glucose and pyruvate were omitted; this medium was named Free-CAP-HEPES-TYH and notated with the symbol (–) in Figure 1. Finally, rescue was performed by incubating sperm first in FREE-HEPES-TYH until motility stopped and then recovered in CAP-HEPES-TYH medium. Following the same abbreviation used throughout this manuscript, this procedure was abbreviated as SER in Figure 1.

To conduct computer-assisted sperm analyses (CASA), for each replicate, cauda epididymides from two mice were dissected, cut in half, and distributed in specific media as follows: (1) for non-capacitating conditions (NON-CAP), two half epididymis from each mouse were collected in NON-CAP-HEPES-TYH containing energy substrates but lacking NaHCO_3_ and BSA; (2) for capacitating conditions (CAP), two half epididymis from each mouse were collected in CAP-HEPES-TYH containing energy substrates and supplemented with 15 mM NaHCO_3_ and 5 mg/mL of BSA; (3 and 4) the remaining half epididymides were collected in FREE-CAP-HEPES-TYH medium devoid of glucose and pyruvate supplemented with 15 mM NaHCO_3_ and 5 mg/mL of BSA. In all these cases, after 10 min of sperm swim-out, epididymides were removed and samples were washed by two sequential centrifugations, using the respective incubation media. Sperm were then counted for each condition; equal number of sperm (∼2 million sperm) were then divided in four aliquots (notice that starved sperm in FREE-CAP-HEPES-TYH are divided in two different aliquots) and incubated at 37°C in 500 μL of their respective medium. After ∼40 min (when sperm in starvation conditions stop moving), to facilitate washing, additional 1.5 mL of the appropriate media (as defined in the next sentence) was added to each aliquot. Appropriate media mean: for condition 1 (NON-CAP), NON-CAP-HEPES-TYH; for condition 2 (CAP), CAP-HEPES-TYH, for condition 3 (–), FREE-CAP-HEPES-TYH; and for condition 4 (SER), same as for condition 2, CAP-HEPES-TYH. Sperm suspensions were then centrifuged for 5 min at 150 × *g* at room temperature, 1.7 mL of supernatants was removed leaving the bottom 300 μL in the tubes to avoid losing sperm cells. Additional 200 μL of the respective media was added and each aliquot incubated for 60 additional minutes. After this period, sperm suspensions (25 μL) were loaded into pre-warmed chamber slide (depth, 100 μm) (Leja slide, Spectrum Technologies) and placed on a microscope stage at 37°C. Sperm motility was examined using the CEROS computer-assisted semen analysis (CASA) system (Hamilton Thorne Research, Beverly, MA, United States) as previously described (Sanchez-Cardenas et al., 2018). The default settings include the following: frames acquired: 90; frame rate: 60 Hz; minimum cell size: 4 pixels; static head size: 0.13–2.43; static head intensity: 0.10–1.52; and static head elongation: 5–100. Sperm with hyperactivated motility, defined as motility with high amplitude thrashing patterns and short distance of travel, were sorted and analyzed using the CASAnova software (Goodson et al., 2011). At least five microscopy fields corresponding to a minimum of 200 sperm were analyzed for each treatment in each experiment.

### Sperm Preparation for *in vitro* Fertilization (IVF)

Media used for IVF assay did not contain HEPES; instead pH 7.4 was maintained using HCO_3_^–^/CO_2_ buffer system. For these assays, we used Toyoda–Yokoyama–Hosi Complete TYH (C-TYH) medium (Toyoda et al., 1971) containing 119.37 mM NaCl, 4.7 mM KCl, 1.71 mM CaCl_2_.2H_2_O, 1.2 mM KH_2_PO_4_, 1.2 mM MgSO_4_. 7H_2_O, 25.1 mM NaHCO_3_, 0.51 mM Na-pyruvate, 5.56 mM glucose, and 4 mg/mL BSA, 10 μg/mL gentamicin and phenol red 0.0006% equilibrated at 5% CO_2_ to reach pH 7.4. For media used for starvation, glucose and pyruvate were omitted from the medium described above and was called F-TYH.

For IVF, spermatozoa were collected from the cauda epididymis. In each case, the two epididymides from the same mouse were separated and incubated in different conditions. One of the cauda was placed in 2 mL of C-TYH, containing glucose and pyruvate and abbreviated as CAP in Figures 2–5. The other one was placed in 2 mL of F-TYH media, not containing nutrients; sperm in this medium will then be rescued and the treatment abbreviated SER in Figures 2–5. After 10 min of sperm swim-out, each cauda epididymis was removed from the tubes, and sperm were washed by two sequential centrifugations using the respective incubation medium as follows. First, sperm suspensions were centrifuged for 5 min at 300 × *g* at room temperature. After that, supernatants were removed, and sperm were re-suspended in 2 mL of C-TYH for the control, and 2 mL of F-TYH for the SER treatment. Samples were centrifuged for additional 5 min at 150 × *g* at room temperature, supernatants were removed, and sperm re-suspended in 500 μL of C-TYH for the CAP control and 500 μL of F-TYH for the SER treatment. Both sperm suspensions were incubated at 5% CO_2_ at 37°C until sperm from F-TYH stopped moving (∼40 min). Then, 1.5 mL of C-TYH was added to both CAP- and SER-treated sperm. Sperm suspensions were then centrifuged for 5 min at 150 × *g* at room temperature and 1.7 mL of supernatants was removed leaving the bottom 300 μL in the tubes. To maintain the initial sperm concentration, additional 200 μL of C-TYH was added to both samples.

### Ca^2+^ Ionophore Treatment for IVF

Ca^2+^ ionophore 4Br-A_23187_ was used at a final concentration of 20 μM in C-TYH as previously described (Navarrete et al., 2016). Briefly, sperm were incubated with Ca^2+^ ionophore 4Br-A_23187_ for 10 min in 5% CO_2_ at 37°C, and subsequently washed twice by mild 5 min centrifugations (150 × *g*) in C-TYH media. For SER and Ca^2+^ ionophore A_23187_ combination, SER treatment was done as explained above with minor modifications. At 35 min of incubation in F-TYH, just before sperm stopped their movement, Ca^2+^ ionophore 4Br-A_23187_ was added for 5 min. Next, 1.5 mL of C-TYH was added and the sperm suspension was centrifuged for 5 min at 150 × *g* at room temperature. Supernatant was removed, and 2 mL of C-TYH was added. Then, the sperm sample was centrifuged once more for 5 min at 150 × *g* and the supernatant removed leaving the bottom 300 μL in the tube. Additional 200 μL of C-TYH was added to reach a final volume of 500 μL.

### Sperm Motility Video Recordings

Sperm were incubated in F-TYH or C-TYH as described above. The respective sperm suspensions (25 μL) were loaded into glass-bottom culture dishes (MatTek Corp., Ashland, MA, United States). Videos were captured using NIS-Elements software on a Nikon TE300 inverted microscope (Chiyoda, Tokyo, Japan) fitted with 20X objective lenses (Plan Apo, NA 0.75) and equipped with a cMOS camera (Andor Zyla, Belfast, Northern Ireland). Temperature of the samples was maintained at 37°C using a stage warmer (Frank E. Fryer scientific instruments, Carpentersville, IL, United States).

### *In vitro* Fertilization

Metaphase II-arrested mouse oocytes were collected from 6–8-week-old super ovulated CD-1 (ICR) or C57BL/6J female mice (Charles River Laboratories, Wilmington, MA, United States) as previously described (Navarrete et al., 2015). Each female was injected with 7.5–10 IU PMSG and hCG 48 h apart. Super-ovulated females were then sacrificed 13 h post-hCG injection, oviducts were dissected, and cumulus-oocyte complexes (COCs) were collected in TL-HEPES medium [containing 114 mM NaCl, 3.22 mM KCl, 2.04 mM CaCl_2_.2H_2_O, 0.35 mM NaH_2_PO_4_.2H_2_O, 0.49 mM MgCl_2_.6H_2_O, 2.02 mM NaHCO_3_, 10 mM Lactic acid (sodium salt), and 10.1 mM Hepes]. Then, COCs were washed in C-TYH medium, and subsequently placed into a 90 μL drop of C-TYH covered with mineral oil previously equilibrated in an incubator with 5% CO_2_ at 37°C. Fertilization drops containing two to three COCs (∼40–60 eggs) were inseminated with 100,000 sperm incubated in different conditions as described above. Fertilization dishes were kept in an incubator with 5% CO_2_ at 37°C. After 4 h of insemination, eggs were washed, put in fresh C-TYH media, and incubated overnight in 5% CO_2_ at 37°C. Fertilization rates (in percentage) were evaluated 24 h post-insemination and were calculated as the total number of two-cell embryos divided by the total number of inseminated oocytes multiplied by 100.

### Embryo Culture and Embryo Transfer

Twenty-four hours post-fertilization, two-cell embryos were transferred to drops containing KSOM media and further incubated for 3.5 days in 5% CO_2_ at 37°C. At this stage, the percentage of blastocyst formation was evaluated. In some cases, 12–18 blastocysts were transferred to 2.5 days post-coitum (dpc) pseudo-pregnant CD-1 recipient females using the non-surgical uterine embryo transfer device NSET (Bin Ali et al., 2014). The number of blastocysts for transfer was selected considering the minimum number of blastocysts available which in all cases corresponded to those obtained from sperm incubated in standard capacitation media. The pseudo-pregnant CD-1 recipient females were obtained by mating with vasectomized males (obtained from Charles River) 2.5 days before the embryo transfers were performed. Only female mice with a visible plug were chosen as embryo recipients.

### Blastocyst Nuclear Staining

A total cell count of 35 individual 3.5-day blastocysts for each condition (control or SER treatment) was performed. Embryos were removed from culture and washed two times in 100 μL drops of PBS containing 1 mg/mL BSA. Then, embryos were fixed in a 100-μL drop of paraformaldehyde solution [4% (w/v) in PBS, pH 7.4] for 20 min at room temperature and washed three times in 100 μL PBS-BSA. After that, embryos were transferred to a 100-μL drop of 1 μg/mL Hoechst 33342 (ThermoFisher Scientific, Agawam, MA, United States) in PBS-BSA and incubated for 10 min at room temperature. Then, embryos were washed two times in 100-μL drops of PBS-BSA and mounted on slides.

Images were taken using an A1 HD25 resonant scanning confocal microscope (Nikon) equipped with an sCMOS camera using a 40X (Plan Fluor, NA 1.3) objective. For each embryo, 10–12 z-stacks of 0.9 μm were taken, and cell number was calculated as the total number of nuclei stained per embryo.

### Blastocysts Outgrowth Assay

Blastocysts were collected and transferred gently into a culture plate coated with 0.1% gelatin (Sigma–Aldrich, St. Louis, MO, United States) and cultured in DMEM (Lonza, Allendale, NJ, United States) containing 10% fetal calf serum (Atlanta Biologicals, Flowery Branch, GA, United States) and 1X GlutaMAX (Thermo Fisher Scientific, Agawam, MA, United States). Outgrowth assays were conducted at 37°C in a humidified atmosphere of 5% CO_2_ for 3 days and were observed daily. Blastocysts outgrowth was considered positive when: (1) they were able to hatch out of the zona pellucida by 24 h, (2) they had attached to the culture plate by 48 h, and (3) they had formed inner cell mass (ICM) colonies with surrounding trophoblast cells at 72 h.

### Bovine Sperm Preparation and SER Treatment

Bovine sperm were obtained from frozen semen samples kindly donated by American Breeder Services (DeForest, WI, United States). Sperm containing straws were thawed at 38°C for 1 min. After that, sperm were separated using a Percoll gradient (45%/90%) by centrifugation 600 × *g* for 15 min.

After Percoll centrifugation, sperm (5 × 10^6^ spermatozoa/mL) were washed in 3 mL of either Sp-TALP [containing 100 mM NaCl, 3.1 mM KCl, 2.04 mM CaCl_2_.2H_2_O, 0.35 mM NaH_2_PO_4_.2H_2_O, 0.4 mM MgCl_2_.6H_2_O, 25 mM NaHCO_3_, 21.6 mM lactic acid (sodium salt), and 1 mM pyruvate] (Parrish et al., 1988) or pyruvate-lactate-free Sp-TALP (free-Sp-TALP) medium, and centrifuged at 150 × *g* for 5 min at room temperature. After that, supernatants were removed, and sperm were re-suspended in 0.5 mL of Sp-TALP for the control, and 0.5 mL of free-Sp-TALP for the SER treatment. Both sperm suspensions were incubated at 5% CO_2_ at 38°C until sperm from free-Sp-TALP showed a considerable reduction in their motility (∼3 h). Then, 1.5 mL of Sp-TALP was added to both control and SER-treated sperm. The samples were then centrifuged for 5 min at 150 × *g* at room temperature, 1.7 mL of supernatants was removed leaving the bottom 300 μL. Sperm were used for ICSI as explained below.

### Bovine Intracytoplasmic Sperm Injection

Intracytoplasmic sperm injection was carried out as previously described (Kurokawa and Fissore, 2003), according to standard protocols, using Narishige manipulators (Medical System Corp., Great Neck, NY, United States) mounted on a Nikon diaphot microscope (Nikon Inc., Garden City, NY, United States). Before ICSI, oocytes were denuded of granulosa cells by gently pipetting in the presence of 1 mg/mL of hyaluronidase and selected by the presence of the first polar body. All matured oocytes were randomly allocated to groups. One part of the sperm suspension was mixed with one part of PBS containing 10% polyvinylpyrrolidone (PVP, M.W. 360 kDa; Sigma). Bovine sperm were immobilized by applying a few piezo pulses to the sperm tail, and the whole sperm was injected. Sperm were delivered into the oocyte’s cytosol using a piezo micropipette-driving unit (Piezodrill; Burleigh Instruments Inc., Rochester, NY, United States).

### Statistical Analysis

Data from all experiments were analyzed using SIGMA plot software^1^. Data are expressed as the means ± SEM. The difference between groups mean values was analyzed by one-way analysis of variance (ANOVA) followed by Tukey’s test. *P*-values < 0.05 were considered significant, and statistical significances were indicated in the figure legends.

## Data Availability Statement

All datasets generated for this study are included in the article/Supplementary Material.

## Ethics Statement

The animal study was reviewed and approved by the University of Massachusetts, Amherst Institutional Animal Care and Use Committee (IACUC), protocol 2016-0026.

## Author Contributions

FN, MG, and PV were responsible for the organization and design of the whole work, data analysis, and preparation of manuscript. FN, LA, DM-H, DT, GL, GA, FG-V, and MG performed the experiments. AS, LL, JB, MB, AD, JM, MG, RF, and PV contributed with experimental design, animal protocols, discussion of findings, and correction of the manuscript.

## Conflict of Interest

LL and JB report owning equity interest in CEP Biotech which has licensed commercialization of a panel of monoclonal antibodies directed against sAC. The method described in this submission is the subject of intellectual property filed by the University of Massachusetts Amherst. It was fully funded by the National Institutes of Health and is subject to rights retained by the United States Government. AS is the founder of Sperm Capacitation Technologies Inc., which is seeking to commercialize the technology. PV is a scientific advisor to the company. The remaining authors declare that the research was conducted in the absence of any commercial or financial relationships that could be construed as a potential conflict of interest.

## Footnotes

1 www.sigmaplot.com

## Supplementary Material

FIGURE S1 Sperm energy restriction and recovery (SER) treatment improves fertilization and embryo development rates in C57BL/6J male mice of different ages. Sperm obtained from C57BL/6J mice of different ages were incubated in C-TYH, containing glucose and pyruvate (CAP) or in F-TYH media, not containing nutrients; sperm in this medium will then be rescued by energy sources replenishment as explained in Methods, and the treatment abbreviated (SER). After treatment (CAP or SER) sperm from each condition were added to the insemination drops containing cumulus-enclosed female oocytes (COCs). In all cases, left panels represent percentages of fertilization calculated as the percentage of oocytes that reached 2-cell embryo stage. Middle panels represent percentages of blastocyst formation calculated as percentage of the 2-cell embryos that reached a 3.5-day blastocyst stage. Right panels represent percentages of total blastocysts calculated as the percentage of 3.5-day blastocyst stage embryos out of the total number of inseminated eggs. **(A–C)** Results from IVF and embryo cultures after heterologous fertilization between CD1 female oocytes and 2–6 months old C57BL/6J sperm (**A**, *n* = 18); 7–12 months old C57BL/6J sperm (**B**, *n* = 10); or 13–24 months old C57BL/6J sperm (**C**, *n* = 10). **(D–F)** Results from IVF and embryo culture after homologous fertilization between C57BL/6J female oocytes and 2–6 months old C57BL/6J sperm (**D**, *n* = 5); 7–12 months old C57BL/6J sperm (**E**, *n* = 5); or 13–24 months old C57BL/6J sperm (**F**, *n* = 5). In each panel, statistically significant differences are indicated as: ^∗^*p* < 0.05; ^∗∗^*p* < 0.01, and ^∗∗∗^*p* < 0.001.

TABLE S1 Compilation of IVF, embryo development, and pups born after embryo transfer.

VIDEO S1 Sperm energy restriction and recovery (SER) treatment. Sperm were incubated in F-TYH at 5% CO_2_ at 37°C until stopped moving (∼40 min). Then, an aliquot was loaded into glass-bottom culture dishes and filmed in an inverted microscope as explained in Methods. C-TYH was added after 10 sec of recording and sperm regained motility.
