# Supplementary figures and images for: Transient Sperm Starvation Improves the Outcome of Assisted Reproductive Technologies
Source: Front Cell Dev Biol. 2019 Nov 5;7:262. doi: 10.3389/fcell.2019.00262 (PMC6848031; doi:10.3389/fcell.2019.00262)

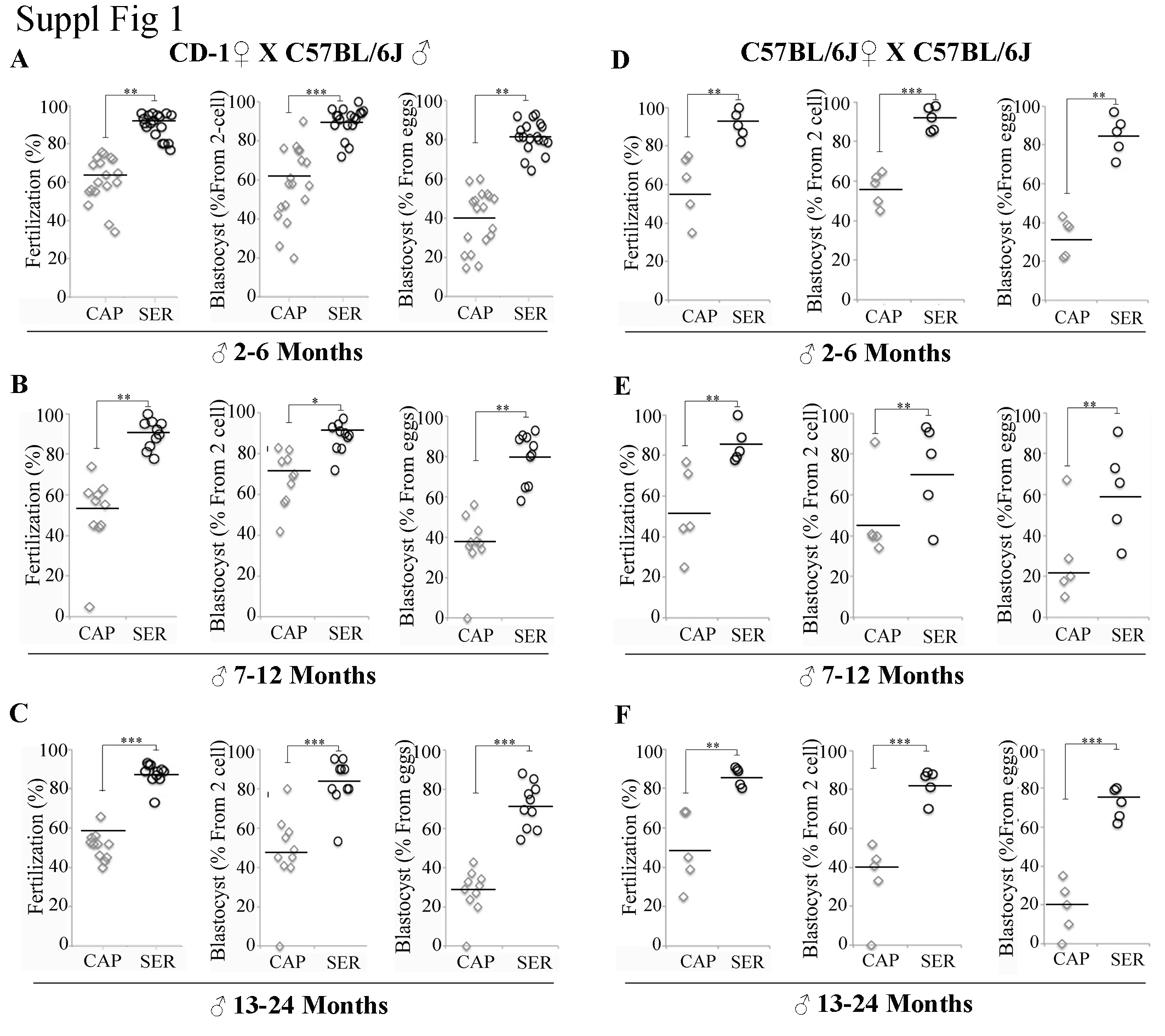

Supplement: FIGURE S1 — Sperm energy restriction and recovery (SER) treatment improves fertilization and embryo development rates in C57BL/6J male mice of different ages. Sperm obtained from C57BL/6J mice of different ages were incubated in C-TYH, containing glucose and pyruvate (CAP) or in F-TYH media, not containing nutrients; sperm in this medium will then be rescued by energy sources replenishment as explained in Methods, and the treatment abbreviated (SER). After treatment (CAP or SER) sperm from each condition were added to the insemination drops containing cumulus-enclosed female oocytes (COCs). In all cases, left panels represent percentages of fertilization calculated as the percentage of oocytes that reached 2-cell embryo stage. Middle panels represent percentages of blastocyst formation calculated as percentage of the 2-cell embryos that reached a 3.5-day blastocyst stage. Right panels represent percentages of total blastocysts calculated as the percentage of 3.5-day blastocyst stage embryos out of the total number of inseminated eggs. (A–C) Results from IVF and embryo cultures after heterologous fertilization between CD1 female oocytes and 2–6 months old C57BL/6J sperm (A, n = 18); 7–12 months old C57BL/6J sperm (B, n = 10); or 13–24 months old C57BL/6J sperm (C, n = 10). (D–F) Results from IVF and embryo culture after homologous fertilization between C57BL/6J female oocytes and 2–6 months old C57BL/6J sperm (D, n = 5); 7–12 months old C57BL/6J sperm (E, n = 5); or 13–24 months old C57BL/6J sperm (F, n = 5). In each panel, statistically significant differences are indicated as: ∗p < 0.05; ∗∗p < 0.01, and ∗∗∗p < 0.001. [file Image_1.TIF]
