# Supplementary material for: Transient Sperm Starvation Improves the Outcome of Assisted Reproductive Technologies
Source: Front Cell Dev Biol. 2019 Nov 5;7:262. doi: 10.3389/fcell.2019.00262 (PMC6848031; doi:10.3389/fcell.2019.00262)
Supplement: TABLE S1 — Compilation of IVF, embryo development, and pups born after embryo transfer. [file Table_1.DOCX]

| **Strain (male x female)** | **Age of the male (months)** | **Sperm**  **treatment** | **Total oocytes (n)** | **n of 2-cell stage (%)** | **n of blastocyst stage (% out of 2-cell)** | **% blastocyst out of oocytes** | **# of embryo transfers (ET)** | **n of ET blastocysts** | **n of pups (% out of transferred blastocysts)** |
| --- | --- | --- | --- | --- | --- | --- | --- | --- | --- |
| **C57BL/6J x CD-1** | 2-6 | CAP | 742 (18) | 486 (65.4) | 299 (62) | 40.2 | 6 | 98 | 18 (18.4) |
|  |  | SER | 880 (18) | 809 (92.0) | 719 (89.1) | 82.3 | 6 | 98 | 58 (59.2) |
|  | 7-12 | CAP | 550 (10) | 280 (50.9) | 203 (72.5) | 36.9 | 3 | 45 | 2 (4.4) |
|  |  | SER | 588 (10) | 528 (89.8) | 466 (88.3) | 79.3 | 3 | 45 | 15 (33.3) |
|  | 13-24 | CAP | 738 (10) | 439 (59.0) | 212 (48.3) | 29.6 | 6 | 93 | 9 (9.7) |
|  |  | SER | 556 (10) | 478 (86.0) | 406 (84.9) | 73.0 | 6 | 93 | 31 (33.3) |
| **C57BL/6J x C57BL6/J** | 2-6 | CAP | 267 (5) | 149 (55.8) | 84 (56.4) | 31.5 | 2 | 34 | 1 (2.9) |
|  |  | SER | 291 (5) | 265 (91.1) | 245 (92.5) | 84.2 | 2 | 34 | 9 (26.5) |
|  | 7-12 | CAP | 197 (5) | 104 (52.8) | 47 (45.2) | 23.9 | 2 | 24 | 7 (29.2) |
|  |  | SER | 169 (5) | 141 (83.4) | 100 (70.9) | 59.2 | 2 | 24 | 11 (45.8) |
|  | 13-24 | CAP | 144 (5) | 74 (51.4) | 30 (40.5) | 20.8 | -- | -- | -- |
|  |  | SER | 225 (5) | 200 (88.9) | 169 (84.5) | 75.1 | -- | -- | -- |

**Supplemental table 1.** Compilation of IVF, embryo development, and pups born after embryo transfer.
